# Wearable Nocturnal Autonomic and Sleep Biomarkers for Predicting Next-Day Headache and Identifying Nociplastic Pain in Patients with Migraine

**DOI:** 10.3390/jcm15103802

**Published:** 2026-05-15

**Authors:** Lewis E. Tomalin, Benjamin R. Kummer, Maya C. Campbell, Asala Erekat, Laura Wandner, Fred Cohen, Daniel Clauw, Jessica Robinson-Papp, Bridget R. Mueller

**Affiliations:** 1 Department of Population Health and Science Policy, Icahn School of Medicine at Mount Sinai, New York, NY 10029, USA; ltomalin@natera.com; 2 Department of Neurology, Icahn School of Medicine at Mount Sinai, New York, NY 10029, USA; benjamin.kummer@mountsinai.org (B.R.K.); maya.campbell@mssm.edu (M.C.C.); asala.erekat@mssm.edu (A.E.); jessica.robinson-papp@mssm.edu (J.R.-P.); 3 Windreich Department of Artificial Intelligence and Human Health, Icahn School of Medicine at Mount Sinai, New York, NY 10029, USA; 4 Clinical Neuroinformatics Center, Icahn School of Medicine at Mount Sinai, New York, NY 10029, USA; 5 David S. and Ruth L. Gottesman Center for Headache Treatment and Translational Research, Icahn School of Medicine at Mount Sinai, New York, NY 10029, USA; lwandner@gmail.com (L.W.); fred.cohen@mssm.edu (F.C.); 6 Chronic Pain and Fatigue Research Center, Department of Anesthesiology, University of Michigan Medical School, Ann Arbor, MI 48109, USA; dclauw@umich.edu

**Keywords:** smartwatch, sleep, autonomic nervous system, electrodermal activity, migraine

## Abstract

**Background/Objectives**: The aim of this pilot study was to evaluate the feasibility of developing individualized machine learning models using nocturnal wearable-derived autonomic nervous system (ANS) and sleep metrics to predict next-day headache risk in patients with migraine. We also examined the associations between nocturnal ANS and sleep measures and patient-reported outcome measures (PROMs) related to nociplastic pain, migraine burden, and non-restorative sleep (NRS). **Methods**: Adults with migraine wore the wrist-worn Empatica EmbracePlus^®^ wearable during sleep and completed daily headache diaries for approximately 4 weeks (N = 10). Participants also completed daily headache diaries and PROMs assessing nociplastic pain, migraine burden, and non-restorative sleep. Personalized machine learning (ML) models were developed to predict next-day headache using nocturnal ANS activity (e.g., pulse rate variability (PRV), electrodermal activity (EDA), respiratory rate (RR)) and sleep metrics (e.g., interruptions, duration, awakenings). Model performance was evaluated using area under the receiver operating characteristic and precision–recall curves (AUROC, AUPRC), sensitivity, specificity, accuracy, and precision. Spearman correlations assessed the relationship between wearable-derived metrics and patient-reported outcome measurements of sleep quality (PROMIS-Fatigue, PROMIS-Sleep Disturbance) and a surrogate marker of nociplastic pain (Fibromyalgia (FM) Score). **Results:** 9 out of 10 participants wore the EmbracePlus device for at least the target duration of four weeks. For the next-day headache prediction, model performance varied between individuals; area under the ROC curve (AUROC) ranged from 28.2% to 81.2%. Nocturnal measures of EDA were strongly correlated with the FM score (Spearman’s rho = 0.72–0.75, *p* < 0.05). **Conclusions**: Phasic EDA may warrant further investigation as a potential physiological indicator related to nociplastic pain mechanisms and next-day headache. However, these findings are preliminary, and larger multicenter trials are needed to confirm results of this pilot study.

## 1. Introduction

Migraine is a prevalent and debilitating neurological disorder affecting more than 40 million Americans. Attacks are characterized by moderate to severe headache often accompanied by sensory hypersensitivity, including photophobia, phonophobia, osmophobia, and nausea [1]. Despite recent advances in migraine therapies that target the trigeminovascular complex (TVC), and inflammatory neuropeptides such as calcitonin gene-related peptide (CGRP), treatment remains challenging for many patients. While the reasons for suboptimal treatment responses are multifactorial, the unpredictable timing of attacks and dysregulated central sensory processing pathways beyond the TVC are increasingly recognized as important contributors [2,3]. Central changes in sensory processing can lead to nociplastic pain, defined by the International Association for the Study of Pain (IASP) as pain that arises from altered nociception despite no clear evidence of actual or threatened tissue damage and has recently been associated with disabling non-painful symptoms, such as orthostatic intolerance and multisensory sensitivity, in some patients with migraine [2,3,4]. Therefore, the present pilot study explores physiological changes preceding migraine attacks and their potential links to nociplastic pain.

There is growing evidence that the activity of the autonomic nervous system (ANS), the body’s primary rapid stress response system, may be altered in the hours prior to migraine onset [5,6], and is *persistently* altered in patients with nociplastic pain [7,8]. These observations highlight the importance of the study of the ANS in patients with migraine. However, the context-dependent nature of ANS responses [9], the complexity of its activity which involves both the sympathetic nervous system (SNS) [9] and parasympathetic/vagal branch [10], and substantial inter-individual variability have contributed to inconsistencies in the literature and limited our understanding of the role of the ANS in migraine and nociplastic pain mechanisms [8,11].

Wearable biosensor technologies offer new opportunities to address these challenges by enabling continuous, real-world monitoring of physiological signals reflective of SNS and parasympathetic activity, as well as objective measures of sleep activity. Nocturnal monitoring is especially important for individuals with migraine, as reduced sleep quality is a well-established risk factor for attacks [12,13,14]. By pairing wearable-derived measurements with personalized machine learning (ML) modeling approaches, the highly individualized ANS responses can be better assessed. However, concerns exist regarding feasibility of the long-term monitoring needed to effectively train ML models in a population with significant sensory sensitivity [15,16].

In this pilot study, we pursued three objectives to provide preliminary data and inform the design of larger wearable-based studies in patients with migraine. First, we assessed the tolerability of at least 4 weeks of wearable nocturnal monitoring in individuals recruited from a tertiary academic headache center. Second, we used nocturnal ANS and sleep metrics to develop individualized ML models that assessed vulnerability of next-day headache. Finally, we sought to identify the specific wearable-derived measurements that were most closely related to measures of nociplastic pain, with the goal of identifying primary outcome selection in future larger studies across more diverse patient populations.

## 2. Materials and Methods

### 2.1. Study Population

Eligible patients were prospectively recruited from the David S. and Ruth L. Gottesman Center for Headache Treatment and Translational Research, Icahn School of Medicine at Mount Sinai (ISMMS), New York, between November 2023 and May 2025. The sample size of 10 participants was selected based on the proof-of-concept nature of this feasibility study involving intensive longitudinal wearable monitoring and individualized machine learning and aligns with several prior pilot and feasibility studies using wearable sensors and machine learning for migraine attack prediction [17,18]. This pilot cohort enabled detailed manual review and iterative refinement of phasic electrodermal activity (EDA) feature extraction, as well as systematic comparison of multiple phasic EDA metrics to identify the most stable and interpretable candidates for future larger-scale validation studies. A formal statistical power analysis was not performed, as the primary analytic approach relied on person-specific models rather than traditional group-level inferential statistics. All participants were required to be 18 years of age or older and met the International Classification of Headache Disorders, 3rd edition (ICHD-3) [19] criteria for migraine as determined by a headache specialist based on at least three months of headache diary data. Exclusion criteria included the presence of another headache or facial pain disorder, or a medical condition or treatment that would confound interpretation of wearable data (e.g., pacemaker), a treatment with known influence on the ANS (beta-blockers, vagal nerve stimulators, stimulants), sleep-related disorder, or pregnancy. Headache days were defined as any day on which participants reported the presence of headache. Although headaches in migraine patients are often considered part of a single migraine spectrum with varying severity and features, we did not use structured symptom diaries to apply formal ICHD-3 criteria and therefore analyzed all self-reported headache days as a single category [19,20]. This binary design prioritizes ecological validity while acknowledging potential phenotypic heterogeneity among headache days. All study procedures were conducted in accordance with a protocol approved by the ISMMS Institutional Review Board, and written informed consent was obtained from all participants.

### 2.2. Study Design

Following consent, participants received an EmbracePlus ^®^ wearable, which was connected to the participants’ smartphone via Bluetooth and configured to stream real-time data into a secure, HIPAA compliant, cloud-based web dashboard (Empatica Health Monitoring Platform). Given known influences of sex-hormones on the ANS, a target of 4 weeks was chosen to include both luteal and follicular phases of the menstrual cycle. The Empatica EmbracePlus ^®^ wearable was chosen as it records electrodermal activity (EDA), a measure of post-ganglionic sympathetic activity with a sampling frequency (4 Hz) that permits reliable separation of the phasic, stimulus-responsive phasic EDA component (closely linked to sympathetic arousal and stress), from the slower thermoregulatory tonic component [21,22,23]. Using a photoplethysmography sensor, thermometer, accelerometer, and gyroscope the Empatica also collects skin temperature (1 Hz), blood volume pulse (64 Hz), systolic peaks (64 Hz), and linear acceleration (64 Hz). Empatica provides both raw data and 1 min aggregated data for skin temperature, sleep detection, body position, EDA, pulse rate variability (PRV), pulse rate (PR), respiratory rate (RR), and accelerometry data. Duration of time in bed, asleep, awake, and time spent out of bed (all measured in minutes) are determined using ANS, body position, and actigraphy indices [24]. Participants tracked headaches using their preferred diary system (paper or electronic). Headache days were defined as any day on which participants reported the presence of headache Although headaches in migraine patients are often considered part of a single migraine spectrum with varying severity and features, we did not use structured symptom diaries to apply formal ICHD-3 criteria and therefore analyzed all self-reported headache days as a single category [16,17]. This binary design prioritizes ecological validity while acknowledging potential phenotypic heterogeneity among headache days. Participants also completed the Migraine Disability Assessment (MIDAS), PROMIS-29 v.2 which assesses 7 health domains including measures of non-restorative sleep (i.e., fatigue and sleep disturbance) [25] and the 2011 Fibromyalgia (FM) Survey Criteria (FM Score), a measure of nociplastic pain [26,27]. The FM Score includes a widespread pain index (WPI) and a symptom severity (SS) scale that when combined can be used to calculate a score ranging from 0 to 31.

### 2.3. Comparison of ANS and Sleep Features Between Nights Preceding Days with and Without Headache

Linear mixed-effects models (LMM) compared nightly physiological measures between nights prior to no headache and headache. For the LMM, data analysis for sleep metrics and ANS indices were performed on 5 min blocks that were derived by averaging 5 consecutive 1 min buckets of biomarker data. Each participant was modeled with a random intercept to account for multiple nights of data from the same individual. However, for phasic EDA analysis, the raw 4 Hz EDA data was analyzed to preserve the temporal resolution required for peak detection and storm duration measurement. This structure adjusts for within-person correlation across repeated measurements and yields group-level estimates of physiological differences between nights. ANS activity recorded during out-of-bed periods (identified using actigraphy) was excluded from analysis to minimize the confounding effects of physical activity on ANS measurement.

### 2.4. Next-Day Headache Prediction

Individualized machine learning models were developed for each participant to predict the probability of next-day headache using nocturnal wearable-derived autonomic and sleep metrics. Raw data were segmented into non-overlapping 5 min blocks during in-bed periods [17]. For each 5 min block, the median, minimum, maximum, and standard deviation were calculated for nine metrics: total EDA, skin temperature, pulse rate (PR), pulse rate variability (PRV), respiratory rate (RR), time in bed, time asleep, time awake in bed, and time spent out of bed. ANS activity during out-of-bed periods (identified via actigraphy) was excluded to minimize confounding by physical activity. Model predictions for next-day headache probability were generated for each valid 5 min interval and then averaged across the night to produce a single nightly prediction score. Three modeling approaches were evaluated: elastic-net regression (for regularization and interpretability), random forests (for capturing nonlinear interactions), and gradient boosting machines (for sequential optimization).

Models were trained and evaluated using a nested cross-validation framework performed at the night level to preserve temporal ordering and prevent information leakage from repeated 5 min intervals within the same night. In the outer loop, a leave-one-night-out procedure was used for unbiased performance evaluation: one entire night was held out as the test set, while the remaining nights were used for training and hyperparameter tuning. Within each outer training set, hyperparameters were optimized using a custom balanced leave-two-nights-out inner cross-validation. In each inner fold, all 5 min intervals from one headache night and one non-headache night were held out together, and all possible balanced pairs were evaluated exhaustively. Hyperparameters were selected to maximize the AUPRC in the inner loop. After tuning, the final model was retrained on the full outer training set and evaluated on the held-out test night.

The analytic dataset contained no missing predictor values. All predictors were centered and scaled prior to model fitting. No wrapper- or filter-based feature selection procedure was applied. Regularization was incorporated inherently through elastic-net penalization, while random forests and gradient boosting machines relied on model-specific hyperparameter tuning within the nested framework. Inverse-frequency class weights were applied during training to address the within-participant class imbalance between headache and non-headache nights. To further mitigate overfitting risk in this small-sample individualized setting, night-level splitting ensured that all 5 min blocks from the same night remained together in the same split. Full details of the nested cross-validation procedure, including a schematic overview (Supplemental Figure S1), are provided in the Supplemental Methods.

### 2.5. Tonic and Phasic EDA Analysis

**Figure 1:**
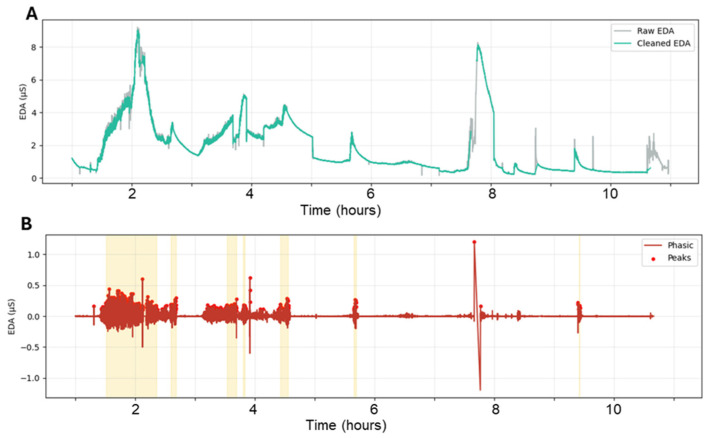
EDA preprocessing, SCR peak detection, and storm identification: (**A**) Representation nocturnal electrodermal activity (EDA) trace before (gray) and after (teal) data processing, which involved removing out-of-bed periods, artifact masking, outliers, interpolation of brief dropouts, removal of short spurious segments, and detrending. (**B**) Phasic EDA component derived from the cleaned signal. SCR peaks denoted by red circle. Yellow shaded regions indicate storm intervals identified using the epoch-based definition in which a 30-s epoch containing at least 3 SCR peaks was considered positive and adjacent positive epochs separated by ≤5 min were treated as a single storm.

Raw nocturnal EDA data were used for tonic-phasic decomposition to preserve the temporal resolution required for peak detection and storm analysis (see Figure 1). SCR peaks were identified using the NeuroKit2 algorithm (see Supplement Methods for algorithm), which detects candidate peaks based on local trough-to-peak dynamics [28]. NeuroKit2 is a widely used open-source physiological signal-processing framework with validated analysis pipelines and has also been used in prior studies analyzing wearable EDA data [29]. In sensitivity analyses, only peaks with a minimum trough-to-peak amplitude of 0.005 µS were retained, and consecutive accepted peaks were required to be separated by at least 1 s [30]. EDA storms were quantified by an epoch-based definition (i.e., 30 s period with at least 3 SCR peaks), as previously described [31].

### 2.6. Statistical Analyses

Demographic, autonomic, and questionnaire data for continuous variables were summarized as means ± standard deviations or medians with interquartile ranges, as appropriate. Categorical variables are reported as counts and percentages. To compare nightly autonomic and sleep measures between nights with and without headache, we used linear mixed-effects models (LMM) that accounted for the repeated observations within each participant. These analyses were exploratory and were not adjusted for multiple comparisons. Night served as the unit of analysis, with a random intercept for participant to handle within-subject correlation. Variables showing clear right skew or heteroscedasticity were log(1 + x)-transformed before modeling; others were analyzed on the original scale. Fixed effects were evaluated with Type III tests, using the Kenward–Roger approximation for denominator degrees of freedom when available (otherwise Satterthwaite). For each LMM, we report the model-estimated difference between headache and no-headache nights with 95% confidence intervals; for outcomes modeled on the log(1 + x) scale, estimates are reported on the transformed scale. We examined associations using Spearman’s rank correlation (ρ) between (1) wearable nocturnal autonomic/sleep measures and (2) established markers of nociplastic pain and non-restorative sleep. Nightly wearable measures were averaged over the duration of testing at the participant level before correlation testing. Two-sided *p*-values are reported without correction for multiple comparisons, in keeping with the exploratory goal of comprehensively evaluating ANS and sleep metrics for future study.

## 3. Results

### 3.1. Study Population Demographics and Clinical Characteristics

Ten participants (8 female, 2 male) with a median age of 45 years of age (IQR, 34–52) were enrolled. The median duration of migraine was 25.0 years (IQR, 10.0–29.0). Five participants met criteria for chronic migraine and 7 reported migraines with aura. Most participants reported using prescription treatments for prevention (9/10) and all reported using prescription acute treatments for migraine (Table 1). Anxiety was the most commonly reported medical comorbidity (Supplemental Table S1). The median MIDAS score was 19.0 (IQR, 8.8–48.0), reflecting moderate migraine-related disability. The median FM score was 11.0 (IQR, 6.5–12.8), indicating a high burden of nociplastic pain near the fibromyalgia threshold of 12. Participants reported mild-to-moderate fatigue (median PROMIS Fatigue T-score 55.1 [IQR, 51.5–67.4]) and mild sleep disturbance (median PROMIS Sleep Disturbance T-score 56.1 [IQR, 48.9–61.7]), both elevated relative to the general population mean of 50.

### 3.2. Empatica EmbracePlus Tolerability and Data Collection Efficiency

**Figure 2:**
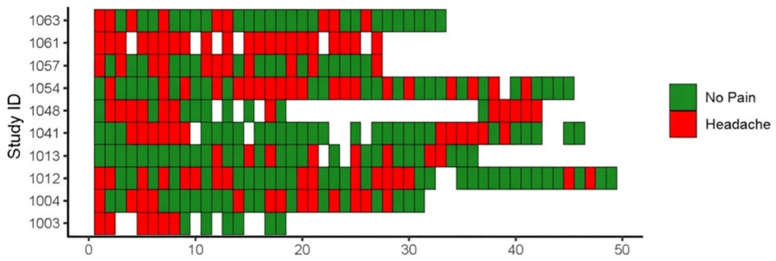
Pictorial representation of headache diary and wearable data collection success. Tile map showing nights on the *x*-axis and study ID on the *y*-axis. Each tile is colored according to the participant’s headache status (no pain vs. headache) on the following day. White spaces indicate days where no watch data was recorded due to data missingness exceeding 30%.

Overall, 9 out of 10 participants wore the EmbracePlus device for at least the target duration of four weeks. One participant reported discomfort and discontinued 10 days prior to planned study termination. Six participants requested to continue wearing the watch beyond the planned 4-week period because they found the real-time biomarker feedback provided through the Empatica platform informative. A total of 316 nights of data with missingness < 30% and a diary entry were collected. Overall, 183 nights preceded headache-free days, and 133 nights preceded days with headache (Table 1 and Figure 2). Participants spent a mean of 8.5 h in bed per night (SD, 1.8; range, 3.4–25.0) and slept for 7.5 h (SD, 1.4; range, 2.9–18.0). Participants wore the device an average of 36.5 nights (SD, 9.9 days). Data collection efficiency, defined as the proportion of worn nights with data missingness < 30%, exceeded 75% (8 out of 10 participants), with a mean of 86.5% (SD, 17.1%; range, 50–100%) (Table 1).

### 3.3. Group-Level Comparison of ANS and Sleep Metrics Preceding Days with No Headache and Headache

**Figure 3:**
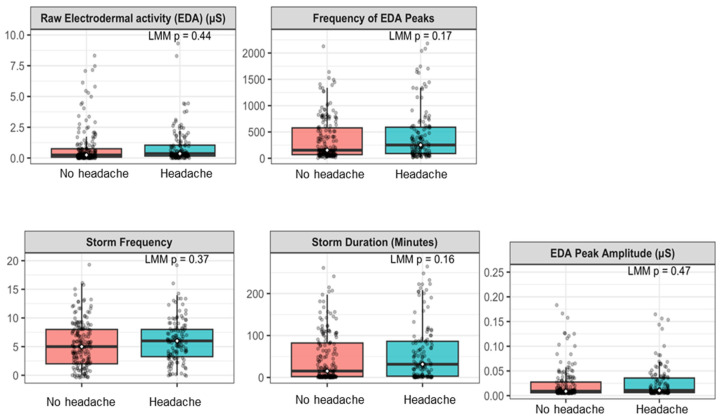
Nocturnal phasic electrodermal activity (EDA) metrics on nights preceding days with versus without headache. Boxplots display total EDA, frequency of EDA peaks, EDA peak amplitude, storm frequency, and storm duration. Points represent individual nights. *p*-values were obtained from linear mixed-effects models (LMM) with a random intercept for participants.

Night-level physiological and sleep measures were compared between nights preceding days with no headache versus headache using linear mixed-effects models with a random intercept. Sleep measures, and cardiovascular ANS measures did not significantly differ between nights preceding days with and without headache (Supplemental Table S2). Among EDA-related measures, median total EDA, frequency of EDA peaks, storm frequency, and storm duration were higher on nights preceding headache compared with nights preceding no headache; however, these differences were not statistically significant. As shown in Figure 3, several phasic measures trended higher on nights preceding headache days, indicating its promise for a threshold-based feature for next-day headache. Although none of the differences reached statistical significance, the LMM estimates corresponded to small-to-moderate effect sizes for the phasic EDA measures (Hedges’ g ranging approximately 0.25–0.50 based on the observed differences and model variance; see Supplemental Table S2 for unstandardized estimates and 95% CIs). These magnitudes are consistent with small-to-medium effects using pain-research-specific interpretive guidelines [32].

### 3.4. Patient-Specific Machine Learning (ML) Prediction of Next-Day Headache

Participant-specific machine learning models for predicting next-day headache were developed and evaluated for 9 of the 10 participants (Table 2A). One participant (ID 1061) was excluded because all monitored nights were followed by a headache day. Model performance varied substantially across participants, with AUROC values ranging from 0.28 to 0.81, and AUPRC values ranging from 0.19 to 0.76. The best-performing model was observed in participant 1054 using a random forest algorithm, achieving an AUROC of 0.812 and AUPRC of 0.760 at a headache prevalence of 45.5%. In contrast, the poorest performance was seen in participant 1063 using Elastic-Net regression (AUROC 0.282, AUPRC 0.189; headache prevalence 27.3%). Using predefined interpretive benchmarks, only participant 1054 met criteria for potentially clinically informative prediction (AUROC ≥ 0.70 and AUPRC ≥ 0.10 above prevalence). For this participant, model performance was further examined across probability thresholds of 25%, 50%, and 75% (Table 2B). A 50% threshold provided the most balanced performance, with both sensitivity and specificity of 75%.

### 3.5. Correlation with Measures of Nociplastic Pain and Non-Restorative Sleep (NRS)

We examined Spearman rank correlations between participant-level averages of nocturnal wearable-derived ANS and sleep metrics and clinical patient-reported outcomes, including the Widespread Pain Index (WPI), Fibromyalgia Survey Score (FM score) as a proxy for nociplastic pain burden, Migraine Disability Assessment Scale (MIDAS), PROMIS Fatigue T-score, and PROMIS Sleep Disturbance T-score.

**Figure 4:**
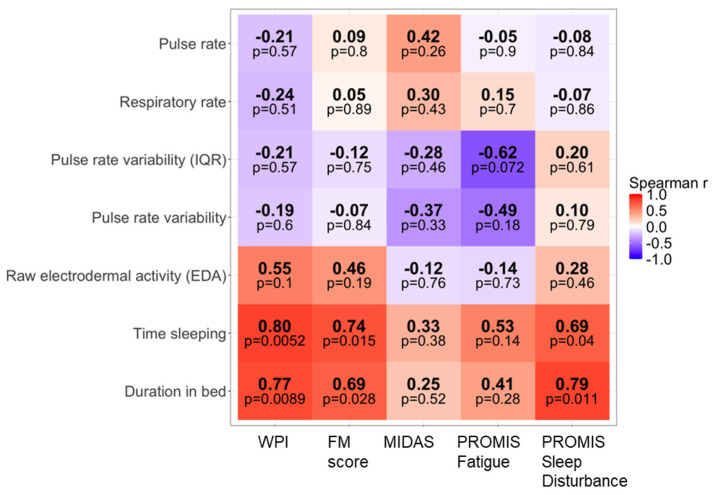
Correlation heatmap of nocturnal sleep-related and non-phasic autonomic measures with patient-reported outcomes. Heatmap displaying Spearman’s rank correlation coefficients (*p*) between participant-level summaries of sleep parameters and non-phasic autonomic nervous system (ANS) metrics derived from the Empatica Embrace Plus wearable and key patient-reported outcomes: Widespread Pain Index (WPI), Fibromyalgia Survey Score (FM score), Migraine Disability Assessment Scale (MIDAS), PROMIS Fatigue T-score, and PROMIS Sleep Disturbance T-score. Spearman’s *p* values are shown in each tile, with corresponding two-sided *p*-values displayed below. All wearable metrics were aggregated at the participant level prior to analysis. No adjustment for multiple comparisons was performed, consistent with the exploratory nature of the study.

Longer total sleep time and time in bed showed large positive correlations with higher WPI, higher FM score, and greater sleep disturbance (Spearman’s ρ = 0.69–0.80, *p* < 0.05; Figure 4). According to pain-research-specific interpretation guidelines, these correlation coefficients reflect large effects. [32] These associations are consistent with the possibility that prolonged but potentially non-restorative sleep may relate to migraine chronification and nociplastic features.

**Figure 5:**
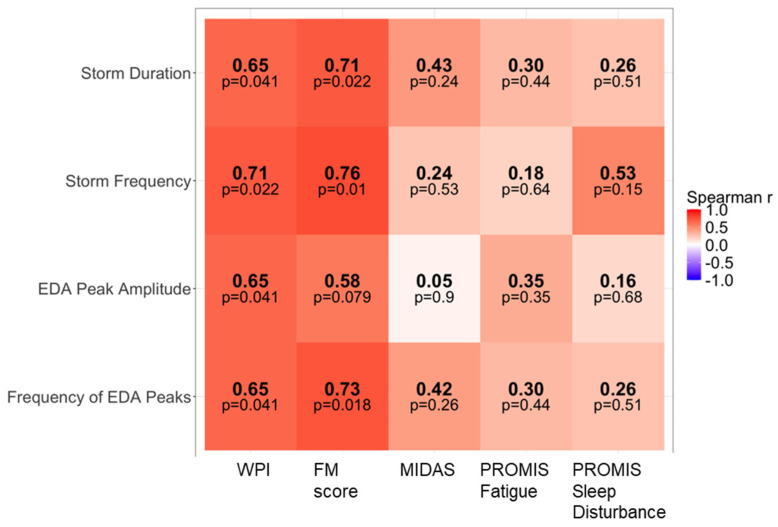
Correlation heat map of phasic EDA-derived measures with patient-reported outcomes. Heat map showing Spearman’s rank correlations between participant-level summaries of phasic electrodermal activity (EDA) measures and patient-reported outcomes, including widespread pain index (WPI), fibromyalgia survey score (FM score), Migraine Disability Assessment Scale (MIDAS), PROMIS Fatigue T-score, and PROMIS Sleep Disturbance T-score. Warmer colors indicate more positive correlations and cooler colors indicate more negative correlations. Values within each tile represent Spearman’s *p*, with corresponding two sided *p*-values shown below. Wearable measures were aggregated at the participant level prior to correlation testing. No adjustment for multiple comparisons was performed, consistent with the exploratory and hypothesis generating nature of the analysis.

Phasic EDA measures also demonstrated positive correlations with markers of nociplastic pain (Figure 5). Frequency of EDA peaks, storm frequency, and storm duration were all positively correlated with both WPI and FM score (Spearman’s ρ = 0.65–0.76, *p* < 0.05) corresponding to medium-to-large effects. EDA peak amplitude was also positively associated with WPI (ρ = 0.65, *p* = 0.041; large effect) and showed a trend with FM score (ρ = 0.58, *p* = 0.079; medium-to-large effect). In contrast, correlations between phasic EDA measures and MIDAS, PROMIS Fatigue, and PROMIS Sleep Disturbance did not reach statistical significance. Given the exploratory and pilot nature of this study, we did not apply correction for multiple comparisons. Approximately 30 Spearman correlation tests were performed across the main variables. Even the strongest observed associations had the lowest *p*-value of 0.01, which would not survive a conservative Bonferroni correction (adjusted α ≈ 0.0017). Therefore, reported correlations should be interpreted cautiously as hypothesis-generating rather than confirmatory.

## 4. Discussion

This pilot study explored nocturnal autonomic nervous system (ANS) activity and sleep patterns in relation to next-day headache and nociplastic pain features in patients with migraine. We also evaluated the tolerability of four weeks of continuous nocturnal wearable monitoring in a population with significant migraine burden.

The EmbracePlus wearable was well tolerated by patients with migraine; 9 out of 10 participants completed at least the target four-week nocturnal monitoring period. This high retention rate supports the feasibility of multi-week ambulatory ANS and sleep monitoring in patients with significant migraine burden.

As anticipated, given the modest sample size and high inter-individual variability in migraine prodrome and ANS physiology, linear mixed-effects models showed no significant group-level differences in averages of ANS and sleep metrics on nights preceding headache and headache-free days [18,33]. Several phasic EDA measures—including SCR peak frequency, EDA storm frequency, and storm duration—were numerically higher on nights preceding headache days than on headache-free nights. While results were not statistically significant and trends should be interpreted with caution, they align with prior work demonstrating an association between sympathetic arousal during sleep and next-day headache vulnerability [6]. Furthermore, the sympathetic neural pathways mediating EDA originates in the hypothalamus, a brain region known to be active during the premonitory, pain-free phase of migraine [34,35].

Individualized machine learning models using multimodal nocturnal ANS and sleep features achieved variable performance in predicting next-day headache. Only one participant’s model reached clinically informative levels (AUROC 81.2%, AUPRC 76.0%), underscoring the challenge of predictive modeling in a clinically heterogeneous cohort which differed in migraine type, duration, comorbidities, and treatments. While reflective of real-world tertiary care, this clinical heterogeneity likely contributed to inter-individual differences in autonomic measures and model performance. Variability in model performance may also have stemmed from our binary outcome of “headache day.” Although headaches in patients with migraine are frequently viewed as part of a migraine spectrum and monthly headache days (MHD) is a common endpoint in migraine research [36], the present study did not employ daily diaries with detailed headache characteristics. Consequently, formal ICHD-3 criteria could not be applied to differentiate migraine days from non-migraine headache days. While this binary design prioritizes ecological validity, it introduces heterogeneity in headache classification and future studies should prioritize identifying patient subgroups and headache phenotypes most likely to benefit from predictive modeling, rather than pursuing broad clinical application at this stage.

We observed strong positive correlations between nocturnal phasic EDA measures and the Fibromyalgia Survey Score (FM score) and Widespread Pain Index (WPI). We used the FM score to capture nociplastic pain features and multisymptomatic distress rather than as a diagnostic tool for fibromyalgia, given that it has not been formally validated as a specific measure of nociplastic pain in migraine populations, and migraine is a primary headache disorder with established neurovascular origins. These medium-to-large associations (Spearman’s ρ = 0.65–0.76) support the hypothesis that persistent sympathetic dysregulation may contribute to nociplastic mechanisms in patients with migraine and identify phasic EDA as a candidate primary outcome for future larger multicenter trials. However, is important to note that statistical significance would not have survived correction for multiple comparisons. Moreover, phasic EDA is a non-specific marker of sympathetic activation and can be influenced by external factors (e.g., caffeine, stress) that were not measured. Thus, larger controlled studies are needed.

Regarding sleep metrics, we found positive correlations between sleep duration/time in bed and measures of nociplastic pain burden. Although unexpected, this pattern is consistent with studies linking non-restorative sleep (NRS) to increased time spent sleeping and time in bed [37,38], suggesting the presence of compensatory sleep behavior. If confirmed, these findings further strengthen the well-documented bidirectional relationship between poor sleep quality and chronic pain [39,40].

This study has several important limitations. First, the small sample size and recruitment from a single tertiary headache center limits generalizability. The high migraine burden in our cohort may not reflect the broader migraine population. Further, the high baseline headache frequency in this pilot cohort may have limited our ability to detect differences between nights preceding headache versus headache-free days in patients with a high frequency of headache, the neurological baseline may be persistently altered, making the delta between nights preceding headache and headache-free days difficult to detect. Accordingly, these findings should be interpreted as preliminary and hypothesis-generating.

Second, several technical limitations of wrist-worn wearable devices should be acknowledged. Pulse rate variability (PRV) derived from photoplethysmography (PPG) was used as a surrogate for ECG-derived heart rate variability (HRV). Although PPG-based PRV has shown strong correlation with ECG-derived HRV in previous validation studies [41,42], it remains more susceptible to motion artifacts and may not fully capture the same physiological information as gold-standard ECG recordings. Similarly, while the NeuroKit2 EDA phasic analysis program has been used in prior wearable studies [29], this method has primarily been validated on laboratory-grade recordings and the signal quality in long-term ambulatory conditions requires further validation. These technical considerations underscore the need for continued refinement of wearable-derived measures.

Third, several variables known to influence ANS activity including caffeine, perceived stress, and menstrual cycle were not measured or controlled on a daily basis, which may have increased model performance variability. Further, although we excluded participants using medications with strong ANS effects (e.g., beta-blockers and stimulants), many used prevention and abortive treatments (i.e., calcitonin-gene-related peptide (CGRP) modulators) that may influence autonomic and vascular physiology. The lack of daily-level diary data on abortive medication and control for these migraine prevention treatments likely introduced variability and influenced model performance.

Fourth, as noted earlier, the FM score has not been formally validated in migraine patients and should therefore be regarded as a non-specific proxy of multisymptom burden rather than a diagnostic tool for fibromyalgia. Finally, because our primary goal was exploratory, we did not apply correction for multiple comparisons, which may have increased the risk of false-positive findings, particularly in the correlational analyses between ANS metrics and patient-reported outcome measures related to nociplastic pain.

## 5. Conclusions

This pilot study supports the feasibility of nocturnal wearable monitoring to capture autonomic signals in patients with migraine. Preliminary, hypothesis-generating results showed phasic EDA measures emerged as promising candidate signals associated with nociplastic pain mechanisms and next-day headache occurrence. However, no causal inferences can be drawn, and these results require confirmation in larger, multicenter studies with rigorous signal validation and control of confounding variables before any clinical application can be considered.

## Figures and Tables

**Table 1:** Clinical and wear-time information for study population.

Study ID	Sex	Age	Years With Migraine	Aura	MIDAS	Nocturnal Data Collection Time, Hours	EmbracePlus Efficiency *
1003	Female	48	35	-	68	8.0 (6.7–9.3)	12/18 (67%)
1004	Female	40	25	+	11	9.1 (8.1–10.1)	31/31 (100%)
1012	Male	25	15	-	53	7.8 (5.8–9.8)	47/49 (96%)
1013	Female	43	27	+	33	10.3 (8.5–12.1)	34/36 (94%)
1041	Female	47	10	+	2	7.0 (6.1–7.9)	39/47 (85%)
1048	Female	37	25	+	N/A	7.1 (6.0–8.2)	21/42 (50%)
1054	Female	23	8	+	8	9.3 (7.8–10.8)	44/45 (98%)
1057	Female	56	38	+	20	8.4 (7.8–9.0)	28/28 (100%)
1061	Male	57	35	-	N/A	9.0 (8.0–10.0)	21/27 (75%)
1063	Female	50	27	+	18	8.3 (7.9–8.7)	33/33 (100%)

* Data presented is median (Q1–Q3), calculated as number of nights with data with missingness < 30%/total nights worn. Abbreviations: MIDAS, Migraine Disability Assessment Scale.

**Table 2:** (**A**) Next-day headache prediction model performance. (**B**) Threshold-based performance of the highest-performing participant (1054) next-day headache model.

(**A**)				
**Study ID**	**Model**	**Headache Prevalence**	**AUROC**	**AUPRC**
1003	Elastic-Net	0.50	0.53	0.62
1004	GBM	0.42	0.46	0.39
1012	GBM	0.36	0.35	0.28
1013	Random Forest	0.24	0.40	0.19
1041	Elastic-Net	0.31	0.68	0.51
1048	GBM	0.57	0.61	0.61
1054	Random Forest	0.46	0.81	0.76
1057	Elastic-Net	0.41	0.57	0.44
1061 *	NA	1.00		
1063	Elastic-Net	0.27	0.28	0.19
(**B**)				
**Threshold**	**Precision**	**Accuracy**	**Sensitivity**	**Specificity**
25%	0.65	0.73	0.85	0.63
50%	0.71	0.75	0.75	0.75
75%	0.73	0.71	0.55	0.83

Abbreviations: AUPRC, area under the precision–recall curve; AUROC, area under the receiver-operating curve; GBM, gradient boosting machine. * patient with no headache-free days, so model could not be computed.

## Data Availability

The original contributions presented in this study are included in the article/Supplementary Material. Further inquiries can be directed to the corresponding authors.

## Supplementary Materials

The following supporting information can be downloaded at: https://www.mdpi.com/article/10.3390/jcm15103802/s1, Supplemental Methods: Machine Learning Workflow and Nested Cross-Validation Framework; Figure S1. Schematic diagram of the machine learning next-day headache prediction modeling: Model performance was evaluated using a leave-one-night-out cross-validation approach. For each iteration, all data from a single night was held out as the test set, while the remaining nights served as the training set. This process was repeated so that each night was used once as the test set. Within each training set, hyperparameters were tuned using leave-two nights-out cross-validation. Each validation fold consisted of one headache night and one no-headache night, and all possible such combinations were evaluated. The model with the optimal hyperparameters from the inner loop was retrained on the full training set and used to make predictions on the held-out test night. Predictions from all test nights were then aggregated to assess the overall performance of the modeling approach. Abbreviations: CV: cross-validation; Table S1. Baseline medical conditions and migraine medication use by participants; Table S2. Night-level autonomic and sleep measures comparing nights preceding headache versus no-headache days.

## Abbreviations

The following abbreviations are used in this manuscript:

ML	Machine Learning
EDA	Electrodermal Activity
PR	Pulse Rate
PRV	Pulse Rate Variability
RR	Respiratory Rate
AUROC	Area Under the Receiver-Operating Curve
AUPRC	Area Under the Precision–Recall Curve
ANS	Autonomic Nervous System
SNS	Sympathetic Nervous System
SCR	Skin Conductance Response
LMM	Linear Mixed-Effects Models
TVC	Trigeminovascular Complex
CGRP	Calcitonin Gene-related Peptide
