# Supplementary material for: Wearable Nocturnal Autonomic and Sleep Biomarkers for Predicting Next-Day Headache and Identifying Nociplastic Pain in Patients with Migraine
Source: J Clin Med. 2026 May 15;15(10):3802. doi: 10.3390/jcm15103802 (PMC13206900; doi:10.3390/jcm15103802)
Supplement: Supplementary file 1 [file jcm-15-03802-s001.zip › jcm-4251016-SI.pdf]

Supplemental Methods

Machine Learning Workflow and Nested Cross-Validation Framework

Individualized machine learning models were developed for each participant to predict next-day headache probability. Data were segmented into non-overlapping 5-minute blocks during detected in-bed periods. For each block, summary statistics (median, minimum, maximum, and standard deviation) were computed for nine metrics: total electrodermal activity (EDA), skin temperature, pulse rate (PR), pulse rate variability (PRV), respiratory rate (RR), time in bed, time asleep, time awake in bed, and time spent out of bed. Blocks containing out-of-bed activity were excluded.

Three models were evaluated: elastic-net regression, random forests, and gradient boosting machines. To ensure robust and reproducible evaluation while preserving temporal structure, a **nested cross-validation framework at the night level** was implemented.

- **Outer loop (performance evaluation)**: A leave-one-night-out (LONO) procedure was used. One entire night served as the independent test set, while the remaining nights formed the training set. This process was repeated until every night had been used once as the test set.
- **Inner loop (hyperparameter tuning)**: Within each outer training set, hyperparameters were tuned using a custom leave-two-nights-out (L2NO) cross-validation. In each inner validation fold, all 5-minute intervals from one headache night and one non-headache night were held out together as the validation set. All possible balanced headache/non-headache night pairs were evaluated exhaustively. Hyperparameters were selected based on the highest area under the precision-recall curve (AUPRC) across inner folds.

After identifying the best hyperparameters in the inner loop, the final model was retrained on the entire outer-loop training set and then evaluated on the held-out test night. Predictions from all test nights were aggregated to compute overall performance metrics (AUROC and AUPRC).

Preprocessing steps included centering and scaling of all predictors. The analytic dataset contained no missing predictor values. No wrapper- or filter-based feature selection was performed. Regularization was handled inherently via elastic-net penalization (through tuning of the α and L1 ratio parameters). For random forests and gradient boosting machines, complexity was controlled through model-specific hyperparameter tuning (e.g., number of trees, maximum depth, learning rate) conducted exclusively within the inner loop. Inverse-frequency class weights were applied during model training to mitigate the effects of within-participant imbalance between headache and non-headache nights.

To reduce the risk of overfitting in this small-sample, individualized setting, the following safeguards were employed: (1) strict night-level data splitting (all 5-minute blocks from the same night remained together), (2) predictor standardization, (3) inherent regularization mechanisms, (4) nested separation of hyperparameter tuning from final evaluation, and (5) class weighting. A schematic diagram of the nested cross-validation framework is provided in Supplemental Figure 1.

All analyses were conducted in R. Elastic-net regression was implemented using *glmnet*, random forests using *randomForest*, and gradient boosting using *xgboost*, with model tuning performed within the prespecified cross-validation framework.

Missing Data Imputation: Missing physiologic data were imputed separately for each patient-night so that values from one participant or one night were not used to impute another. Imputation was restricted to sleep

periods with non-missing pain labels. For each patient-night, time within the night was represented using sine and cosine transforms of minute-of-night, allowing the imputation model to account for circadian position. The variables entered into the imputation model were pulse rate, pulse rate variability (RMSSD), respiratory rate, temperature, electrodermal activity, and step counts, together with the sine and cosine time terms. Imputation used multivariate imputation by chained equations with predictive mean matching. Patient-nights with fewer than 100 fully observed rows across the physiologic variables were not imputed. The resulting single completed dataset was then used for downstream feature extraction and individualized modeling.

EDA Data Preprocessing: Raw EDA was sampled at 4 Hz and preprocessed before tonic-phasic decomposition and SCR peak detection. Analyses were restricted to prespecified normal nocturnal sleep windows. To align EDA with sleep state, only samples occurring during normal windows and classified as asleep (sleep-detection stage 101, excluding wake and interrupted periods) were retained; all other samples were treated as artifact or missing. Statistical outliers were then identified using a centered 30-second rolling median and rolling median absolute deviation, with a Chebyshev-based threshold corresponding to 95% coverage. After masking these samples, brief gaps of up to 3 minutes were interpolated using time-based interpolation. To avoid retaining short isolated valid fragments between larger invalid regions, any nonmissing interval of 10 minutes or less bounded on both sides by masked data was reclassified as missing. The cleaned signal was subsequently partitioned into contiguous nonmissing segments, each of which was median-detrended with a 10-minute window to reduce slow baseline drift before tonic-phasic decomposition and SCR peak detection.

SCR peak detection: SCR peaks were identified from the phasic EDA signal using the NeuroKit2 peak-detection pipeline. Briefly, all local maxima in the phasic signal were first identified as candidate peaks. For each candidate peak, prominence was computed relative to its left and right bases. The left base was defined by traversing leftward from the local maximum until reaching a point of equal or greater amplitude, or the beginning of the signal, and recording the minimum value within that interval. The right base was defined analogously by traversing rightward until reaching a point of equal or greater amplitude, or the end of the signal, and recording the minimum value within that interval. Peak prominence was then calculated as the peak amplitude minus the larger of the two base values. After computing prominence for all candidate peaks, the maximum prominence across the signal was obtained, and candidate peaks with prominence less than 10% of this maximum were excluded. The remaining candidate peaks were retained as SCR peaks. For each accepted peak, the onset was defined as the nearest preceding local minimum, and half-recovery was defined as the first point to the right of the peak at which the signal returned to 50% of the peak amplitude. In sensitivity analyses, only peaks with a minimum trough-to-peak amplitude of 0.005 μS were retained, and consecutive accepted peaks were required to be separated by at least 1 second.

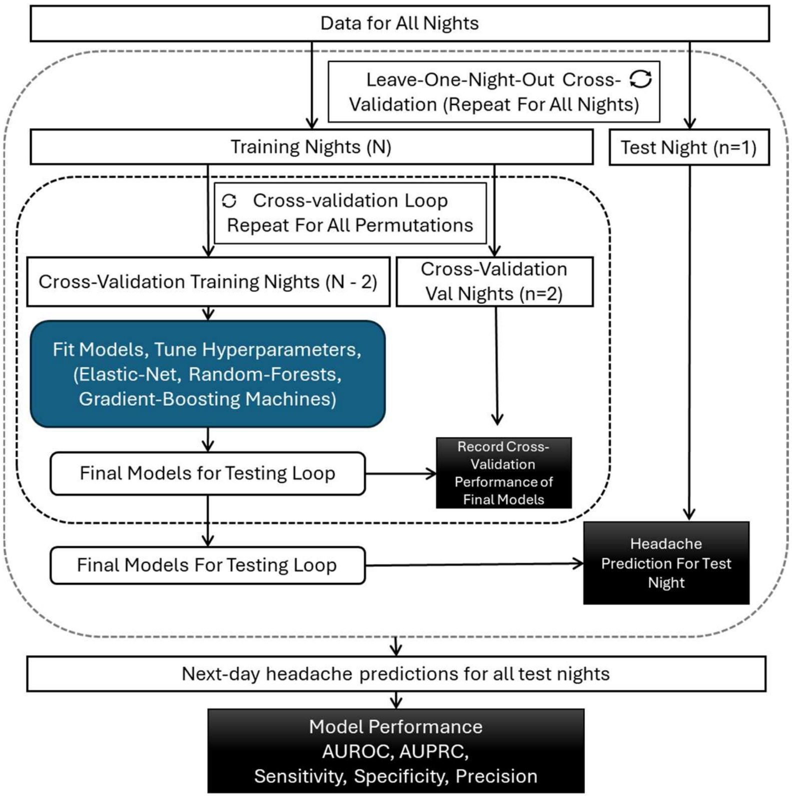

Figure S1. Schematic diagram of the machine learning next-day headache prediction modeling: Model performance was evaluated using a leave-one-night-out cross-validation approach. For each iteration, all data from a single night was held out as the test set, while the remaining nights served as the training set. This process was repeated so that each night was used once as the test set. Within each training set, hyperparameters were tuned using leave-two nights-out cross-validation. Each validation fold consisted of one headache night and one no-headache night, and all possible such combinations were evaluated. The model with the optimal hyperparameters from the inner loop was retrained on the full training set and used to make predictions on the held-out test night. Predictions from all test nights were then aggregated to assess the overall performance of the modeling approach. Abbreviations: CV: cross-validation.

Table S1. Baseline medical conditions and migraine medication use by participants.

ID	Medical Conditions	Migraine Prophylactic	Migraine Abortive
1003	None	Gabapentin onabotulinumtoxinA Galcanezumab-gnlm	Rizatriptan
1004	Depression, Anxiety, Irritable Bowel Syndrome	Atogepant	Rizatriptan
1012	None	None	Advil Ubrogepant
1013	Raynaud's Disease, Hypothyroidism, Fibromyalgia, Irritable Bowel Syndrome, Chronic Fatigue Syndrome	Atogepant onabotulinumtoxinA Gabapentin	Rizatriptan Rimegepant
1041	Asthma, Anxiety	Gabapentin Galcanezumab-gnlm	Ubrogepant
1048	Anxiety, Gastroesophageal Reflux Disease	Gabapentin Galcanezumab-gnlm onabotulinumtoxinA	Rimegepant Advil

1054	Asthma, Depression, Anxiety, Attention Deficit Hyperactivity Disorder	None	Rimegepant
1057	Asthma, Anxiety, Diabetes, Temporomandibular Disorder	Galcanezumab-gnlm	Advil, Ubrogepant
1061	Hyperlipidemia, Irritable Bowel Syndrome, Erectile Dysfunction	Topiramate onabotulinumtoxinA	Ubrogepant, Naproxen
1063	Chronic low back pain	Topiramate onabotulinumtoxinA	Rimegepant

Table S2. Night-level autonomic and sleep measures comparing nights preceding headache versus no-headache days.

	**All (N ** = 316)**	**Night Preceding No Headache (N = 183)**	**Night Preceding Headache (N = 133)**	**Linear Mixed Model (LMM) Estimate (95% CI) ***	**LMM *p*-Value**
Time in bed (minutes)	504.0 [446.8, 563.3]	505.0 [453.0, 566.0]	503 [443.0, 562.5]	3.81 (−18.67, 26.30)	0.74 †
Time sleeping (minutes)	443.0 [397.8, 498.0]	443.0 [398.0, 495.5]	444 [394.0, 501.0]	−3.80 (−21.03, 13.43)	0.67 †
Time awake in bed (minutes)	53.5 [39.0, 72.3]	54.0 [40.5, 73.0]	53.0 [37.0, 72.0]	0.019 (−0.059, 0.097)	0.63 ‡
Pulse rate (beats per minute)	69.8 [65.9, 75.7]	70.4 [66.7, 76.0]	69.3 [64.8, 75.1]	−0.55 (−1.49, 0.39)	0.25 †
Temperature (C°)	34.5 [33.9, 34.9]	34.5 [33.9, 34.9]	34.5 [33.9, 34.8]	−0.11 (−0.22, −0.00)	0.11 †
Pulse rate variability (PRV)	26.7 [17.7, 34.1]	26.7 [18.1, 33.5]	26.7 [17.1, 34.9]	0.040 (−0.026, 0.106)	0.24 ‡
Respiratory Rate (breaths per minute)	16.4 [15.7, 17.3]	16.4 [15.5, 17.4]	16.1 [15.8, 17.20]	−0.12 (−0.34, 0.09)	0.27 †
Total Electrodermal activity (EDA) microsiemens (μS)	0.29 [0.11, 0.97]	0.25 [0.07, 0.75]	0.36 [0.16, 1.05]	0.040 (−0.061, 0.141)	0.44 ‡
EDA Peaks (count)	203.5 [75.0, 587.3]	153.5 [68.3, 577.3]	252.0 [89.3, 589.8]	0.166 (−0.071, 0.403)	0.17 ‡
EDA Peak Amplitude	0.01 [0.01, 0.03]	0.01 [0.01, 0.03]	0.01 [0.01, 0.04]	0.003 (−0.004, 0.009)	0.47 ‡
Storm Frequency	6.0 [3.0, 8.0]	5.0 [2.0, 8.0]	6.0 [3.3, 8.0]	0.058 (−0.068, 0.184)	0.37 ‡
Storm Duration (Minutes)	23.3 [2.4, 83.9]	15.25 [2.0, 82.1]	31.5 [3.0, 86.3]	0.233 (−0.088, 0.555)	0.16 ‡

Data are presented as median [interquartile range] across nights.
* LMM estimate represents the model-estimated difference for headache versus no-headache nights, with 95% confidence intervals; small-to-moderate standardized effects (approximately 0.25–0.50) are inferred for the EDA peaks and storm duration given the observed trends.
**N refers to number of nights.
† Linear mixed-effects model (LMM) fit on the raw outcome.
‡ Linear mixed-effects model (LMM) fit on the log(1 + x) transformed outcome for inference; descriptive statistics are shown in the original units.
